# Prognostic significance of CD163+ tumor-associated macrophages in colorectal cancer

**DOI:** 10.1186/s12957-021-02299-y

**Published:** 2021-06-24

**Authors:** Tao Xue, Kejing Yan, Yiqi Cai, Jiancheng Sun, Zhejing Chen, Xiaolei Chen, Wenyi Wu

**Affiliations:** 1 Department of Traumatology, The First Affiliated Hospital of Wenzhou Medical University, Wenzhou, 325000 Zhejiang Province China; 2 Department of Microbiology and Immunology, School of Basic Medical Sciences, Wenzhou Medical University, Wenzhou, 325035 Zhejiang Province China; 3 Department of Gastrointestinal Surgery, The First Affiliated Hospital of Wenzhou Medical University, No. 1 West Fanhai Road, Ouhai District, Wenzhou, 325000 Zhejiang Province China

**Keywords:** Colorectal cancer, Immunohistochemistry, CD163, Tumor-associated macrophages, Prognosis

## Abstract

**Background:**

This study aimed to explore the prognostic significance of tumor-associated macrophage (TAM) infiltration in colorectal cancer (CRC) patients.

**Methods:**

Tissue microarray and immunohistochemistry were used to detect the infiltration of CD163+ TAMs in 209 CRC samples, and the Kaplan–Meier method was used for survival analysis. Cox proportional hazards analysis was used for univariate analysis and multivariate analysis of clinically relevant confounders.

**Results:**

The samples were divided into low-level (n = 105) and high-level infiltration groups (n = 104) by the median number of CD163+ TAMs detected. The overall survival (OS) and disease-free survival (DFS) of CRC patients in the low-level CD163+ TAM infiltration group were longer than those in the high-level CD163+ TAM infiltration group (P < 0.001). Infiltration of CD163+ TAMs in CRC tissues was a negative prognostic factor for CRC patients. Risks of death and disease recurrence for CRC patients in the low-level CD163+ TAM infiltration group were lower than those in the high-level CD163+ TAM infiltration group (HR_OS_ = 0.183, 95% CI 0.052–0.647, P = 0.008; HR_DFS_ = 0.191, 95% CI 0.078–0.470, P = 0.000).

**Conclusions:**

The infiltration of CD163+ TAMs in CRC tissue is an independent adverse factor for the prognosis of CRC patients. High-level infiltration of CD163+ TAMs is associated with shorter OS and DFS.

**Supplementary Information:**

The online version contains supplementary material available at 10.1186/s12957-021-02299-y.

## Background

There are a variety of immune cells in the tumor microenvironment, including innate immune cells and adaptive immune cells. Numerous studies have shown that these immune cells interact with cancer cells in complex ways [1]. In cancer involving tissues of the digestive system, the levels of macrophages, activated mast cells, and CD4+ memory activated T cells may be higher than in normal tissues [2]. Mast cell infiltration is associated with increased Annexin A1 expression in triple-negative breast cancer [3]. Programmed cell death 1 (PD-1) blockade is effective in immunotherapy of various tumors, but may cause hyperprogressive disease in immunotherapy of advanced gastric cancer [4]. In addition, several studies have been published examining the application of immunotherapy based on the function of immune cells in the tumor microenvironment [5].

Studies have also focused on the relationship between the clinical features, prognosis, and immune therapy of colorectal cancer (CRC) patients and immune cell infiltration of T lymphocytes [6, 7], Programmed death-ligand 1(PD-L1) [8], and CD20+ B lymphocytes [9]. It has also been shown that the infiltration of tumor-associated macrophages (TAMs) is associated with metastasis of CRC [10], and macrophage-derived IL-6 is associated with drug resistance of CRC [11].

Macrophages in the tumor microenvironment are divided into M1 and M2 macrophages [12]. Activated M1 macrophages promote anti-tumor immune responses by regulating antigen presentation and secreting pro-inflammatory cytokines [13]. M2 macrophages promote tumor development by producing anti-inflammatory cytokines [14]. The purpose of this study was to evaluate the relationship between the infiltration of CD163+ TAMs and the clinical features and prognosis of CRC patients.

## Materials and methods

### Patients

For this study, 209 primary CRC surgical specimens from the Second Affiliated Hospital, Wenzhou Medical University, were retrospectively collected from 2001 to 2009. All samples were confirmed to be CRC adenocarcinomas by two pathologists and none of the enrolled patients had received postoperative chemotherapy or other treatments. The clinicopathological features of patients were also collected. TNM stages were classified according to the American Joint Committee on Cancer guideline (7^th^ Edition). Ethical approval for the study was obtained from the ethics committee of the Second Affiliated Hospital, Wenzhou Medical University, and informed consent was also obtained from all patients.

### Tissue microarray and immunohistochemistry

**Fig. 1:**
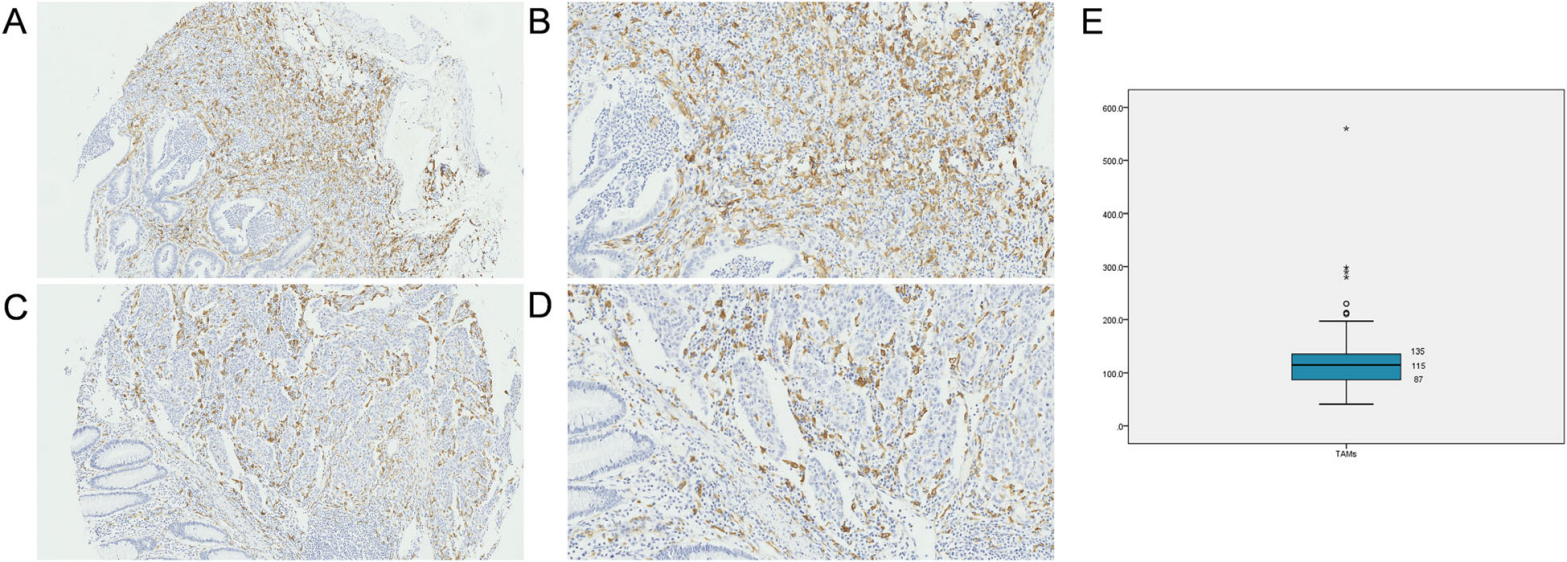
Representative images of staining of CD163+ TAMs. Tumor tissue with high TAM infiltration are shown at 200× (**A**) and 400× (**B**) magnification, and tumor tissue with low TAM infiltration are shown at 200× (**C**) and 400× (**D**). **E** Graphs stratifying patients by CD163+ TAM levels in 209 CRC patients based on a median value of 115

The morphologically representative areas with a diameter of 1 mm were selected following hematoxylin and eosin-staining and were punched from formalin-fixed and paraffin-embedded tissues. All samples were included into one ngTMA block to produce the tissue array using an automated tissue microarrayer (Grandmaster, 3DHistech, Hungary). Each tissue spot included at least 50% tumor cells and used for immunohistochemistry study as previously described [15]. Tissue arrays were incubated with primary antibody for CD163 (ab182422, Abcam) at a dilution of 1:500 at 4 °C overnight after dewaxing, rehydration, and elimination of endogenous peroxidase. Next, the tissues were incubated with the secondary antibody (ab97080, Abcam; at a dilution of 1:2000) at room temperature for 10 min and stained with diaminobenzidine for 1.5 min and counterstained with hematoxylin for 30 s. Positive staining was calculated in three different high-power fields (40× objective) (Fig. 1A). The number of infiltrated CD163+ TAMs was recorded as the mean number of these three values. Two independent pathologists who were blinded to the clinical data evaluated the immunostaining and the results were averaged.

### Clinical parameters of outcomes and statistical basis

The age distribution was transformed as categorical variable, with 60 years as the cut-off value for comparisons. The CEA and CA199 values were also divided into two groups with normal values of 5.0 ng/mL and 37 U/mL as the cut-off values, respectively [16]. The median of the positive numbers of CD163+ TAMs was selected as the cut-off value. Patients were divided into two groups based on the number of examined lymph nodes with a cut-off value of 12 [17]. These data are reported as n (%), and the comparison of clinical characteristics between the low-level TAM group and high-level TAM group was performed by chi-square test. The Kaplan–Meier method was used for survival analysis and the log-rank test for comparison of overall survival (OS) and disease-free survival (DFS) curves. Cox proportional hazard analysis was used for univariate and multivariate analysis for clinically relevant confounders. All statistical analyses were performed using statistical package SPSS (version 22.0 for Windows, IBM SPSS statistics) and P-values < 0.05 were considered statistically significant.

## Results

### Association between CD163+ TAM infiltration and clinical characteristics

According to the median value of CD163+ TAM infiltration, patients were divided into high-level TAMs (> 115) and low-level TAMs (≤ 115) groups (Fig. 1). We analyzed the correlation between CD163+ TAM infiltration in CRC tissues and various clinical characteristics. There were no significant differences in terms of age, sex, TNM stage, location, differentiation, and CEA and CA199 levels between the two groups, with the exception of the number of examined lymph nodes (Table 1).

**Table 1:** Demographic and baseline characteristics in CRC patients with low and high TAM infiltration groups

Characteristics^a^	CD163+ TAMs ≤115 (n = 105)	CD163+ TAMs > 115 (n = 104)	P value^b^
Age (years)			0.607
≤ 60	42 (40.0%)	38 (36.5%)	
> 60	63 (60.0%)	66 (63.5%)	
Sex			0.216
Male	62 (59.0%)	70 (67.3%)	
Female	43 (41.0%)	34 (32.7%)	
Location			0.427
Rectum	75 (71.4%)	69 (66.3%)	
Colon	30 (28.6%)	35 (33.7%)	
TNM stage			0.599
I	71 (67.6%)	68 (65.4%)	
II	27 (25.7%)	25 (24.0%)	
III	7 (6.7%)	11 (10.6%)	
Differentiation			0.081
Poorly	0 (0.0%)	3 (2.9%)	
Moderately	103 (98.1%)	101 (97.1%)	
Well	2 (1.9%)	0 (0.0%)	
Examined lymph nodes			0.020
≤ 12	18 (17.1%)	7 (6.7%)	
> 12	87 (82.9%)	97 (93.3%)	
CEA			0.721
< 5	79 (75.2%)	76 (73.1%)	
≥ 5	26 (24.8%)	28 (26.9%)	
CA199			0.300
< 37	102 (97.1%)	98 (94.2%)	
≥ 37	3 (2.9%)	6 (5.8%)	

*TAMs*, tumor-associated macrophages; *TNM*, tumor node metastasis; *CEA*, carcinoembryonic antigen; *CA*, carbohydrate antigen

^a^n (%)

^b^Chi-square test

### Association between CD163+ TAM infiltration, clinical characteristics, and overall survival

**Fig. 2:**
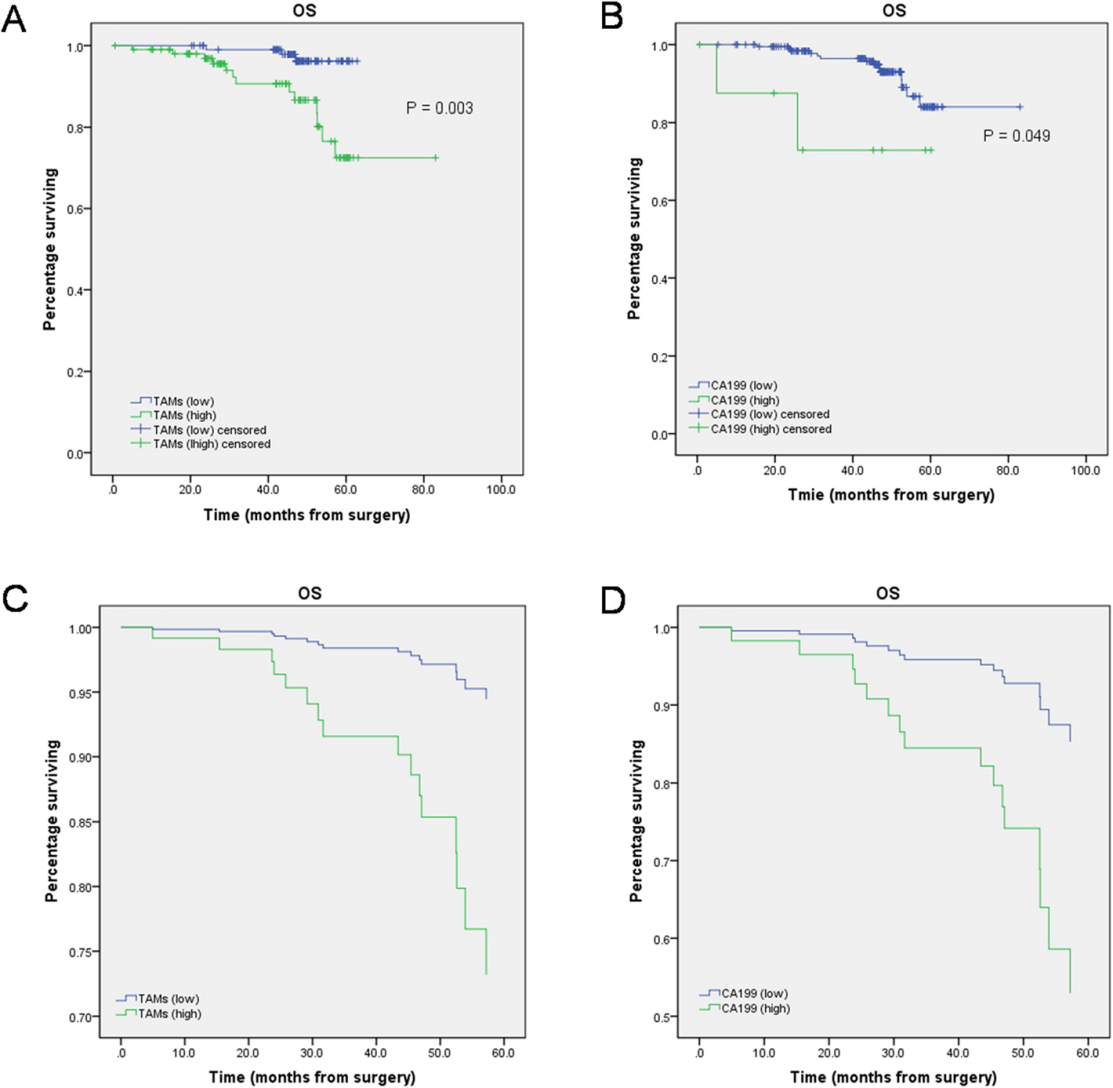
Kaplan–Meier analyses and Cox regression analysis of overall survival (OS) stratified by different TAM infiltration and different serum levels of CA199. **A**, **B** Kaplan–Meier analyses of OS. **C**, **D** Cox regression analysis of OS

We first performed Kaplan–Meier survival analysis to explore the prognostic value of TAM infiltration in CRC tissues. The results showed that the low-level TAM infiltration group (P < 0.001) had a prolonged OS (Fig. 2A), which was confirmed by the results of the univariate Cox regression analysis (Fig. 2C). The results of survival curves for each variable showed that only TAM infiltration and serum level of CA199 were available for Cox regression analysis (Fig. 2A, B). Furthermore, Cox regression analysis showed that TAM infiltration was an independent prognostic predictor of OS (Fig. 2C, D), and CRC patients with low-level TAM infiltration had a lower risk of death than patients with high-level TAM infiltration (HR = 0.183, 95% CI 0.052–0.647, P = 0.008) (Table 2).

**Table 2:** Cox univariate and multivariate analysis for overall survival (OS) stratified for different TAM infiltration

Parameters	Univariate Cox’s regression			Multivariate Cox’s regression		
	P value	HR	95% CI	P value	HR	95% CI
TAMs (low vs. high)	0.008	0.183	0.052–0.647	0.008	0.183	0.052–0.647
CA199 (< 37 vs. ≥ 37)	0.068	0.251	0.057–1.109	-	-	-

*HR*, hazard ratio; *95% CI*, 95% confidence interval; *CA*, carbohydrate antigen

### Association between CD163+ TAM infiltration, clinical characteristics, and disease-free survival

**Fig. 3:**
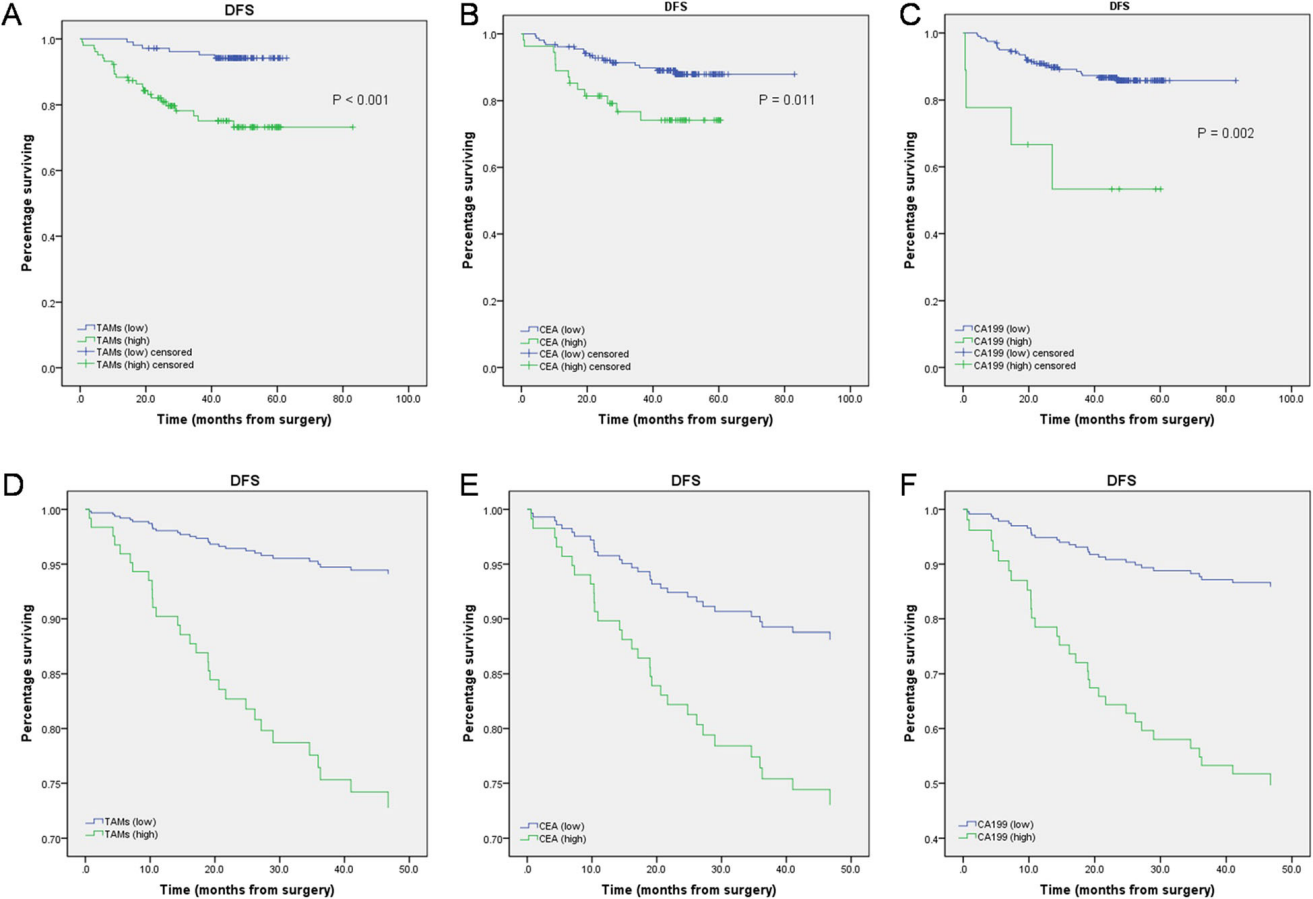
Kaplan–Meier analyses and Cox regression analysis of disease-free survival (DFS) stratified by different levels of TAM infiltration and serum levels of CEA and CA199. **A–C** Kaplan–Meier analyses of DFS. **D**–**F** Cox regression analysis of DFS

The results of Kaplan–Meier survival analysis revealed prolonged DFS in the low-level TAM infiltration group (P < 0.001) (Fig. 3A), which was confirmed by the results of the univariate Cox regression analysis (Fig. 3D). The survival curves for each variable showed that TAM infiltration and serum levels of CEA and of CA199 were suitable for subsequent Cox regression analysis (Fig. 3A–C). TAM infiltration and serum CEA levels revealed to be independent prognostic predictors of DFS (Fig. 3D–F), and CRC patients with a low-level TAM infiltration had a lower risk of recurrence than patients with high-level TAM infiltration (HR = 0.191, 95% CI 0.078–0.470, P < 0.001), low levels of serum CEA were also related to a slower risk of recurrence (HR = 0.403, 95% CI 0.195–0.830, P = 0.014) (Table 3).

**Table 3:** Cox univariate and multivariate analysis for disease-free survival (DFS) stratified for different TAM infiltration

Parameters	Univariate Cox’s regression			Multivariate Cox’s regression		
	P value	HR	95% CI	P value	HR	95% CI
TAMs (low vs. high)	0.000	0.191	0.078–0.470	< 0.001	0.192	0.078–0.473
CEA (< 5 vs. ≥ 5)	0.014	0.403	0.195–0.830	0.030	0.430	0.201–0.920
CA199 (< 37 vs. ≥ 37)	0.005	0.218	0.076–0.625	0.063	0.351	0.116–1.058

*HR*, hazard ratio; *95% CI*, 95% confidence interval; *TAMs*, tumor-associated macrophages; *CEA*, carcinoembryonic antigen; *CA*, carbohydrate antigen

## Discussion

In this study, we evaluated the effects of CD163+ TAM infiltration in CRC tissues on the prognosis of CRC patients. The results suggested that CRC patients with high-level TAM infiltration in CRC tissues had worse prognosis than patients with low-level TAM infiltration. COX regression analysis further demonstrated that the infiltration of CD163+ TAMs in CRC tissues was an independent risk factor for the prognosis of CRC patients, and high-level TAM infiltration in CRC tissues was associated with poorer OS and DFS. In addition, serum levels of CEA also associated with DFS in CRC patients.

The prognosis and metastasis of cancer are related to many factors in the tumor microenvironment, including immune cell infiltration [18], cytokine release [19], protein expression [20], and the interaction between different factors [21, 22]. Experimental studies have shown that high-density TAMs in tumor tissues are associated with low survival rates of breast cancer [23], liver cancer [24], and bladder cancer [25]. However, the relationship between TAM infiltration and the prognosis of CRC patients is still uncertain [26]. Our results revealed that high-level CD163+ TAM infiltration is associated with poor prognosis of CRC patients, which is consistent with the results of Herrera et al. [27]. However, a meta-analysis showed that CD163+ TAM infiltration is independent of the 5-year OS in CRC patients [28], which is inconsistent with the results of this study. This may be related to the different population and having received chemotherapy before surgery.

TAMs are distributed in different microanatomical locations of CRC tissues, such as the center of the tumor and the invasive front of the tumor. TAMs at different locations may include variations in different biological and prognostic characteristics. Wei et al. proposed that an increase in CD163+ TAM infiltration at the invasive front of the tumor is significantly related to the poor prognosis of CRC patients and may play a role in promoting the spread and invasion of CRC [10]. Algars et al*.* showed that the interstitial infiltration of CD163+ TAMs in CRC tissues was associated with a significant increase in survival rates and may exert an anti-tumor role [29]. In contrast, Shabo et al. determined that the density of macrophages in the tumor stroma was related to poor survival [30]. Unfortunately, our study could not distinguish between different areas of the CRC tissue, which could have contributed to the impact and robustness of our results. In addition, in our study, data relative to tumor size and tumor deposits were unavailable and thus we could not perform a relevant analysis.

Furthermore, COX regression analysis showed that the infiltration of CD163+ TAMs was an independent prognostic factor for CRC patients and a risk factor for death and recurrence. The surface antigen CD163 is used for the identification of M2 macrophages, which are considered “bad” macrophages because they participate in Th2 immune response and can release anti-inflammatory cytokines able to promote tumor development [14], although there is evidence that CD163 is not a M2-specific marker [31]. Therefore, whether the relationship between highly infiltrating CD163+ TAMs and poor prognosis of CRC is due to M2 cells is still uncertain, and further research is needed to provide more definitive evidence.

## Conclusions

In summary, our study demonstrates that infiltration of CD163+ TAMs is an independent prognostic factor in patients with CRC. High levels of CD163+ TAM infiltration in CRC tissues is predictive of shorter OS and DFS, which has guiding significance for the prognosis and postoperative treatment of CRC patients undergoing resection surgery.

## Supplementary Information

**Additional file 1:.** Supplementary Figure

## Data Availability

All data generated or analyzed during this study are included in this published article.

## References

[1] Wu T, Dai Y (2017). Tumor microenvironment and therapeutic response. Cancer Lett.

[2] Yang S, Liu T, Cheng Y, Bai Y, Liang G (2019). Immune cell infiltration as a biomarker for the diagnosis and prognosis of digestive system cancer. Cancer Sci.

[3] Okano M, Oshi M, Butash AL, Katsuta E, Tachibana K, Saito K, et al. Triple-negative breast cancer with high levels of annexin A1 expression is associated with mast cell infiltration, inflammation, and angiogenesis. *Int J Mol Sci*. 2019;**20**(**17**). 10.3390/ijms20174197. 10.3390/ijms20174197 PMC6747082 31461932

[4] Xue J, Yu X, Xue L, Ge X, Zhao W, Peng W (2019). Intrinsic beta-catenin signaling suppresses CD8(+) T-cell infiltration in colorectal cancer. Biomed Pharmacother.

[5] Burugu S, Dancsok AR, Nielsen TO (2018). Emerging targets in cancer immunotherapy. Semin Cancer Biol.

[6] Kuwahara T, Hazama S, Suzuki N, Yoshida S, Tomochika S, Nakagami Y, Matsui H, Shindo Y, Kanekiyo S, Tokumitsu Y, Iida M, Tsunedomi R, Takeda S, Yoshino S, Okayama N, Suehiro Y, Yamasaki T, Fujita T, Kawakami Y, Ueno T, Nagano H (2019). Intratumoural-infiltrating CD4 + and FOXP3 + T cells as strong positive predictive markers for the prognosis of resectable colorectal cancer. Br J Cancer.

[7] Zhao Y, Ge X, He J, Cheng Y, Wang Z, Wang J, Sun L (2019). The prognostic value of tumor-infiltrating lymphocytes in colorectal cancer differs by anatomical subsite: a systematic review and meta-analysis. World J Surg Oncol.

[8] Ho HL, Chou TY, Yang SH, Jiang JK, Chen WS, Chao Y, Teng HW (2019). PD-L1 is a double-edged sword in colorectal cancer: the prognostic value of PD-L1 depends on the cell type expressing PD-L1. J Cancer Res Clin Oncol.

[9] Zhang QW, Liu L, Gong CY, Shi HS, Zeng YH, Wang XZ, Zhao YW, Wei YQ (2012). Prognostic significance of tumor-associated macrophages in solid tumor: a meta-analysis of the literature. PLoS One.

[10] Wei C, Yang C, Wang S, Shi D, Zhang C, Lin X, Liu Q, Dou R, Xiong B (2019). Crosstalk between cancer cells and tumor associated macrophages is required for mesenchymal circulating tumor cell-mediated colorectal cancer metastasis. Mol Cancer.

[11] Yin Y, Yao S, Hu Y, Feng Y, Li M, Bian Z, Zhang J, Qin Y, Qi X, Zhou L, Fei B, Zou J, Hua D, Huang Z (2017). The immune-microenvironment confers chemoresistance of colorectal cancer through macrophage-derived IL6. Clin Cancer Res.

[12] Kim J, Bae JS (2016). Tumor-associated macrophages and neutrophils in tumor microenvironment. Mediators Inflamm.

[13] Ostuni R, Kratochvill F, Murray PJ, Natoli G (2015). Macrophages and cancer: from mechanisms to therapeutic implications. Trends Immunol.

[14] Chen Y, Song Y, Du W, Gong L, Chang H, Zou Z (2019). Tumor-associated macrophages: an accomplice in solid tumor progression. J Biomed Sci.

[15] Kristiansen M, Graversen JH, Jacobsen C, Sonne O, Hoffman HJ, Law SK, Moestrup SK (2001). Identification of the haemoglobin scavenger receptor. Nature.

[16] Compton C, Fenoglio-Preiser CM, Pettigrew N, Fielding LP (2000). American Joint Committee on Cancer Prognostic Factors Consensus Conference: Colorectal Working Group. Cancer.

[17] Shulman LN, Browner AE, Palis BE, Mallin K, Kakade S, Carp N, McCabe R, Winchester D, Wong SL, McKellar DP (2019). Compliance with cancer quality measures over time and their association with survival outcomes: The Commission on Cancer’s experience with the quality measure requiring at least 12 regional lymph nodes to be removed and analyzed with colon cancer resections. Ann Surg Oncol.

[18] Yao RR, Li JH, Zhang R, Chen RX, Wang YH (2018). M2-polarized tumor-associated macrophages facilitated migration and epithelial-mesenchymal transition of HCC cells via the TLR4/STAT3 signaling pathway. World J Surg Oncol.

[19] Wada Y, Morine Y, Imura S, Ikemoto T, Saito Y, Takasu C, Yamada S, Shimada M (2020). HIF-1alpha expression in liver metastasis but not primary colorectal cancer is associated with prognosis of patients with colorectal liver metastasis. World J Surg Oncol.

[20] Lin S, Lv Y, Xu J, Mao X, Chen Z, Lu W (2019). Over-expression of Nav1.6 channels is associated with lymph node metastases in colorectal cancer. World J Surg Oncol.

[21] Ding Y, Liu N, Chen M, Xu Y, Fang S, Xiang W, Hua X, Chen G, Zhong Y, Yu H (2021). Overexpressed pseudogene MT1L associated with tumor immune infiltrates and indicates a worse prognosis in BLCA. World J Surg Oncol.

[22] Song D, Wang Y, Zhu K, Tian L, Gao Q, Zhou J, Fan J, Wang X (2020). DCK is a promising prognostic biomarker and correlated with immune infiltrates in hepatocellular carcinoma. World J Surg Oncol.

[23] Yang M, Li Z, Ren M, Li S, Zhang L, Zhang X, Liu F (2018). Stromal infiltration of tumor-associated macrophages conferring poor prognosis of patients with basal-like breast carcinoma. J Cancer.

[24] Yang Y, Ye YC, Chen Y, Zhao JL, Gao CC, Han H, Liu WC, Qin HY (2018). Crosstalk between hepatic tumor cells and macrophages via Wnt/beta-catenin signaling promotes M2-like macrophage polarization and reinforces tumor malignant behaviors. Cell Death Dis.

[25] Martinez VG, Rubio C, Martinez-Fernandez M, Segovia C, Lopez-Calderon F, Garin MI, Teijeira A, Munera-Maravilla E, Varas A, Sacedon R (2017). BMP4 induces M2 macrophage polarization and favors tumor progression in bladder cancer. Clin Cancer Res.

[26] Yang C, Wei C, Wang S, Shi D, Zhang C, Lin X, Dou R, Xiong B (2019). Elevated CD163(+)/CD68(+) ratio at tumor invasive front is closely associated with aggressive phenotype and poor prognosis in colorectal cancer. Int J Biol Sci.

[27] Herrera M, Herrera A, Dominguez G, Silva J, Garcia V, Garcia JM, Gomez I, Soldevilla B, Munoz C, Provencio M (2013). Cancer-associated fibroblast and M2 macrophage markers together predict outcome in colorectal cancer patients. Cancer Sci.

[28] Li J, Li L, Li Y, Long Y, Zhao Q, Ouyang Y, Bao W, Gong K (2020). Tumor-associated macrophage infiltration and prognosis in colorectal cancer: systematic review and meta-analysis. Int J Colorectal Dis.

[29] Nagorsen D, Voigt S, Berg E, Stein H, Thiel E, Loddenkemper C (2007). Tumor-infiltrating macrophages and dendritic cells in human colorectal cancer: relation to local regulatory T cells, systemic T-cell response against tumor-associated antigens and survival. J Transl Med.

[30] Shabo I, Olsson H, Elkarim R, Sun XF, Svanvik J (2014). Macrophage infiltration in tumor stroma is related to tumor cell expression of CD163 in colorectal cancer. Cancer Microenviron.

[31] Barros MH, Hauck F, Dreyer JH, Kempkes B, Niedobitek G (2013). Macrophage polarisation: an immunohistochemical approach for identifying M1 and M2 macrophages. PLoS One.

