# Supplementary figures and images for: Prognostic significance of CD163+ tumor-associated macrophages in colorectal cancer
Source: World J Surg Oncol. 2021 Jun 24;19:186. doi: 10.1186/s12957-021-02299-y (PMC8229299; doi:10.1186/s12957-021-02299-y)

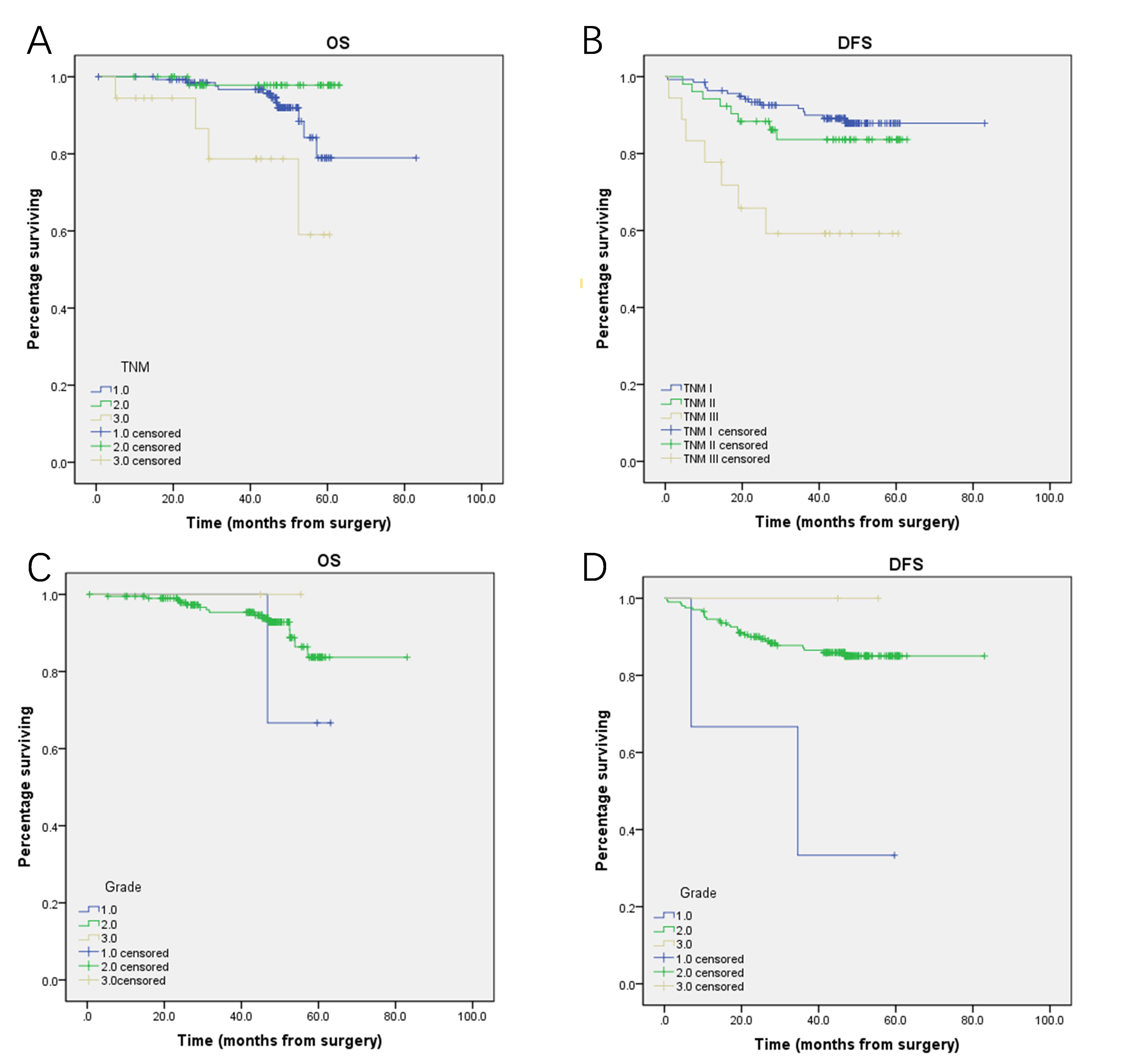

Supplement: Supplementary file 1 — Additional file 1:. Supplementary Figure [file 12957_2021_2299_MOESM1_ESM.tif]
